# An Investigation into the Relationship Between Heart Rate Recovery in Small-Sided Games and Endurance Performance in Male, Semi-professional Soccer Players

**DOI:** 10.1186/s40798-020-00273-8

**Published:** 2020-09-10

**Authors:** Lars Reinhardt, Stephan Schulze, Eduard Kurz, René Schwesig

**Affiliations:** Department of Orthopedic and Trauma Surgery, Martin-Luther-University Halle-Wittenberg, Ernst-Grube-Str. 40, 06120 Halle (Saale), Germany

**Keywords:** Soccer-specific test, Performance diagnostic, Global positioning system, Team sports, Wearable sensor

## Abstract

**Background:**

The ability to recover in the shortest possible time plays an important role especially in intermittent sports such as soccer. Evidence suggests that a well-developed endurance performance has positive effects on the repeated-sprint ability and thus also on the short-term recovery. However, it has not been clarified whether these relationships still exist in a soccer-specific situation. Therefore, the purpose of this investigation was to evaluate the ability of semi-professional soccer players to recover during standardized small-sided games (SSGs) as an endurance performance indicator.

**Methods:**

Eighteen male semi-professional soccer players (age, 23.5 ± 3.7 years) performed an incremental treadmill test (ITT) to determine their running velocity and heart rate at a fixed lactate threshold of 4 mmol L^−1^ (v4). Two days later, the players carried out six bouts of 4 vs. 4 SSGs (duration, 90 s; load to rest ratio, 1:1). A GPS-based tracking system was used to determine distances covered at four fixed speed zones (i.e., < 7.2 km/h, 7.2–14.4 km/h, 14.4–19.8 km/h, > 19.8 km/h) and total distance covered during the SSGs. Furthermore, the frequency of occurrence of accelerations (> 1.54 m s^−2^) was calculated. SSGs’ internal load was quantified by average heart rate and blood lactate concentration after the SSGs. Their recovery ability was evaluated using heart rate recovery (HRR) after the last bout of the SSGs.

**Results:**

A very large correlation (*r* = − .91) with an explained variance of 84% was found between HRR and v4. Further, a better performance in the ITT was also related with a higher number of accelerations executed during SSGs (*r* = .60). The total distance and distances in predefined speed zones did not show any association to v4.

**Conclusions:**

This study showed a strong relationship between HRR after standardized 4 vs. 4 SSGs and the soccer players’ endurance performance in a laboratory setting. Thus, besides being associated with endurance capacity, v4 seems sufficient to evaluate the sport-specific ability to recover in soccer players.

## Key Points

- The running velocity at a fixed lactate threshold of 4 mmol L^−1^ as determined in an incremental treadmill test not only is a strong indicator of endurance performance but also showed a strong association to the ability to recover from soccer-specific activity.
- Small-sided games are suitable as a soccer-specific test situation in order to evaluate the ability to recover.
- In small-sided games, the number of accelerations executed seems to mirror player’s level of endurance.
- A duration of 90 s and a work to rest ratio of 1:1 are a suitable protocol to provoke a very high blood lactate concentration in a soccer-specific environment confirming the potential usefulness of this small-sided game modality to evoke intensity that may improve both aerobic and anaerobic performances.

## Background

Soccer has been commonly defined as an intermittent sport characterized by repetitive movements incorporating frequent bursts of high-intensity activity interspersed with regular recovery periods [1]. Although the total distance covered during official matches in the English Premier League remained nearly constant from 2006 to 2013, there was a 30% increase in the high-intensity (> 19.8 km/h) running distance [2, 3]. As high-intensity runs can be performed only for a short period of time, the frequency of occurrence of high-intensity phases has grown, and therefore, player’s potential to recover from those actions is of increasingly significance [4]. Accordingly, the repeated-sprint ability (RSA) has been shown to be better in professional than in amateur soccer players, despite a similar maximum oxygen uptake (VO_2max_) which is considered as a fundamental prerequisite in soccer [4, 5]. In addition, RSA performance was related to VO_2max_, VO_2_ kinetics, and selected physiological responses, such as blood hydrogen ion concentration, blood bicarbonate concentration, and blood lactate concentration (BLC), in a standardized, high-intensity, intermittent exercise [4]. Typically, an incremental treadmill test (ITT) is performed for determining VO_2max_ [6]. Besides VO_2max_, running velocity at a fixed blood lactate threshold of 4 mmol L^−1^ (v4) is another essential determinant for aerobic endurance performance that can be assessed with an ITT [7–12].

As already mentioned, the ability to recover in the shortest possible time plays an important role especially in intermittent sports such as soccer [4]. To evaluate the level of this capability, several parameters can be calculated across various physical tests [13]. However, measures derived from heart rate (HR), such as HR recovery (HRR), can be easily and efficiently applied in a real context in a feasible way. HRR is defined as the rate at which HR declines immediately after (within a few minutes) the cessation of physical exercise [14]. It involves a coordinated interaction of parasympathetic reactivation and sympathetic withdrawal [15]. Moreover, under standardized laboratory test conditions (e.g., running on a treadmill), HRR has been shown to be positively related to VO_2max_ [14, 15]. However, laboratory tests are functionally limited for soccer, are time consuming, and require high levels of personal and technical support [16].

In recent years, portable player tracking technologies based on global positioning systems (GPS) with integrated accelerometers have become a standard monitoring tool in team sports [17]. This technology allows assessment of physical (distance, velocity, acceleration) and physiological (HR) performance measures during training and competition and opens up the possibility to conduct performance diagnostics under real-world conditions. Therefore, match data can now be evaluated which may represent the most optimal test environment. However, the level of standardization is low, since running performance in soccer is determined by a large number of influencing factors. Determinants like playing position, strategy, tactical orientation, current score, quality of opposition, period of season, match location, or match type play important roles [18, 19]. Although there is no doubt that physical capabilities affect running performance [16, 20, 21], activity profiles during matches do not necessarily display the full physical capabilities of a player.

A possible approach to exclude a majority of the previously mentioned influences is to utilize small-sided games (SSGs) as a standardized, soccer-specific test situation. SSGs, such as the 4 players vs. 4 players, are particularly suitable, since they already represent a widely applied training method at all ages and levels of competition [22]. An essential element of SSGs is that they offer a wide range of variation possibilities in order to achieve different training objectives. By altering game conditions, such as rules, pitch area, number of players, game duration, work to rest ratio, or the presence of goalkeepers, the exercise intensity can be controlled in an effective and sophisticated way [22–24]. With regard to the implementation of SSGs in the soccer-specific performance diagnostic, the main benefits are (i) a standardized load-rest relationship, (ii) the independence of the playing position, and (iii) activity at a consistent high intensity.

The objective of this investigation was to determine the relationships between the outcomes of a standardized ITT (v4) and the physical performance (distances in defined speed zones/acceleration efforts) as well as the physiological response (HR, HRR, and lactate) during SSGs (i.e., 4 vs. 4) in soccer.

## Methods

### Participants and Procedures

Eighteen male semi-professional soccer players (age, 23.5 ± 3.7 years [mean ± SD]; body mass, 78.0 ± 7.3 kg; height, 182.4 ± 7.5 cm; and body mass index, 23.4 ± 1.4 kg/m^2^) from the same team competing in the German 4th league volunteered to participate in this investigation. All healthy and uninjured players of the squad were included in the study, which was conducted in June 2019 during the first week of pre-season preparation. It consisted of two parts within a period of 3 days. On the first day, participants completed an ITT and 48 h later a soccer-specific field test in the form of SSGs.

### Incremental Treadmill Test

The ITT has been carried out on a commercially available running ergometer (Run 260G, Pulse Fitness, Congleton, UK) with the gradient set at 0%, under controlled laboratory conditions (temperature, 22 °C; humidity, 57%). All players were familiar to treadmill running and the ITT, respectively. The test protocols for the ITT and BLC evaluation have been previously published [11]. In short, the initial speed of 7.2 km/h was increased by 1.8 km/h every 3 min (pause duration, 45 s) up to a final speed of 18 km/h. In order to adapt to treadmill running, all subjects performed a 2-min warm-up at a speed of 7.2 km/h prior to the ITT. There was no rest between the warm-up and the first running interval. HR was recorded by the same measuring system as in the SSGs. For determining BLC, capillary blood samples (10 μL) were obtained from the earlobe immediately after each stage of the ITT. BLC was determined by an amperometric enzymatic method (SUPER GL compact, Dr. Müller Gerätebau, Freital, Germany). The velocity and HR at a fixed BLC of 4 mmol L^−1^ in the ITT (HR_v4_, v4) were approximated using Winlactat software version 4.7.0.6 (Mesics, Münster, Germany).

### Small-Sided Games

SSGs were played outdoors on natural turf on a 40 × 30 m pitch with standard goals (2.44 m tall and 7.32 m wide). The objective of the SSGs was to score goals, and the goalkeepers were allowed to use their hands. External encouragement was provided by the technical staff during the realization of the SSGs. A standardized 15-min warm-up consisting of low-intensity running, striding, and dynamic stretching was performed by the participants prior to the SSGs. Additional balls were placed around the pitch in order to maintain game flow and to avoid prolonged rests during the SSGs, thus raising the internal load (HR). A total of three SSGs (goalkeepers included) were performed. Each player took part in one SSG that was composed of six bouts of 90 s in duration separated by 90 s of passive recovery. The players were randomly assigned to the teams. At the beginning of each bout, a 40-m sprint was performed by all players in order to reach a high starting HR. These measurements were carried out on a cloudless, precipitation-free day (temperature, ~ 20 °C; relative humidity, 69%; atmospheric pressure, 1018 hPa; wind speed, 7 km/h).

#### Physical Performance

Positional data and accelerations as well as HR responses were measured using a portable 10-Hz GPS-based tracking system (Polar Team Pro, Polar Electro, Kempele, Finland). The sensors were worn by each player, attached to the skin over the xiphoid process. As recently reported, the measurement errors of the tracking system used for 10- and 20-m sprint times and distances were below 0.18 s or 1.35 m, respectively [25]. Time series of HR, distance, velocity, and acceleration were exported from the Polar Team Pro as CSV files and further analyzed using custom-written programs in MATLAB R2016a (The MathWorks, Natick, MA, USA). Besides the total distance (TD), the analysis of the GPS data was based on the distance covered in four commonly used speed zones [3]: walking (WAL, < 7.2 km/h), jogging (JOG, 7.2–14.4 km/h), running (RUN, 14.4–19.8 km/h), and high-intensity-running (HIR, > 19.8 km/h). Accelerations were quantified as the number of efforts above a fixed threshold of 1.54 m s^−2^ (*n*_acc_) which corresponds to about twice the standard deviation of player’s accelerations in official games (unpublished personal data). Data from all six bouts (excluding rest periods) were summed for further analyses. Only complete datasets without erroneous measurements were included in the final evaluation (14/18).

#### Physiological Response

**Fig. 1:**
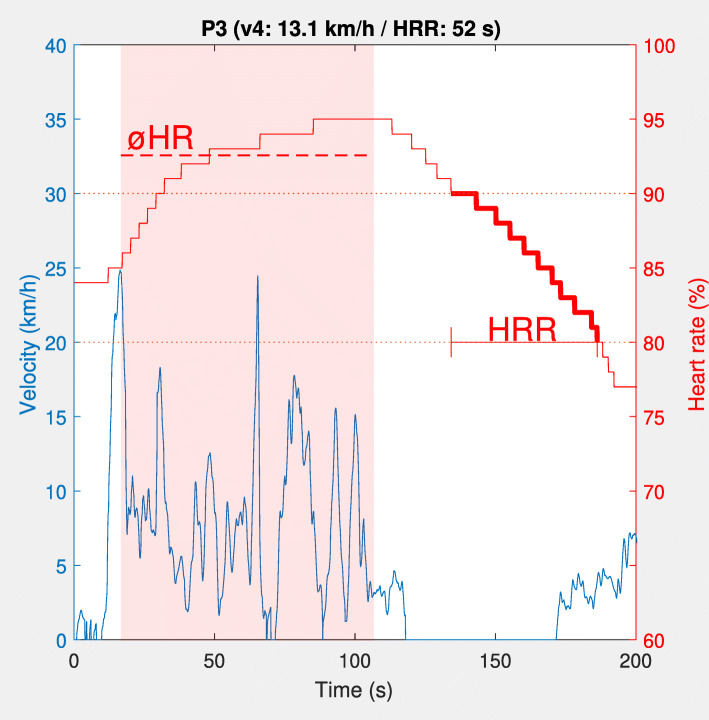
Exemplary time course of velocity (blue curve) and heart rate (as percentage of maximum heart rate, red curve) during the last bout (depicted by a transparent red rectangle) of the small-sided games and the subsequent recovery period. The average heart rate (ØHR) during the playing phase is displayed as dotted red line. The period for determining the heart rate recovery (HRR; decrease in heart rate from 90 to 80%) is indicated in bold

All HR data were normalized to each player’s individual maximum, which was equivalent to the highest reasonable value ever reached during a match or an intensive training session (the team used the Polar Team Pro on a daily basis). The physiological demands during the SSGs were estimated as average HR (ØHR). The ability to recover was determined via the HRR. Parameter HRR was the time (in seconds) necessary to decrease from 90 to 80% after the last SSGs bout (Fig. 1). Based on existing findings by Watson et al. [26], this represents a linear section within the first 90 s of the S-shaped HR curve after a maximum loading. The internal load caused by the SSGs was also evaluated via BLC immediately after the 6th round (L_SSGs_).

### Statistical Analysis

Statistical analyses were performed using IBM SPSS Statistics for Windows (version 23, IBM, Armonk, NY, USA). Results are presented as means ± standard deviations (SDs). Normal distribution of data was checked visually and verified using the Shapiro-Wilk test. Homogeneity of variances was examined using Levene’s test. Pearson’s product moment correlations were carried out to examine linear relationships between selected performance variables. Criteria adopted for interpreting the magnitude of correlations (*r*) between measures were as follows: < 0.2, trivial; 0.2–0.3, small; 0.3–0.5, moderate; 0.5–0.7, large; 0.7–0.9, very large; and 0.9–1.0, almost perfect [27]. Interindividual variability was calculated using the coefficient of variation (CV).

## Results

**Fig. 2:**
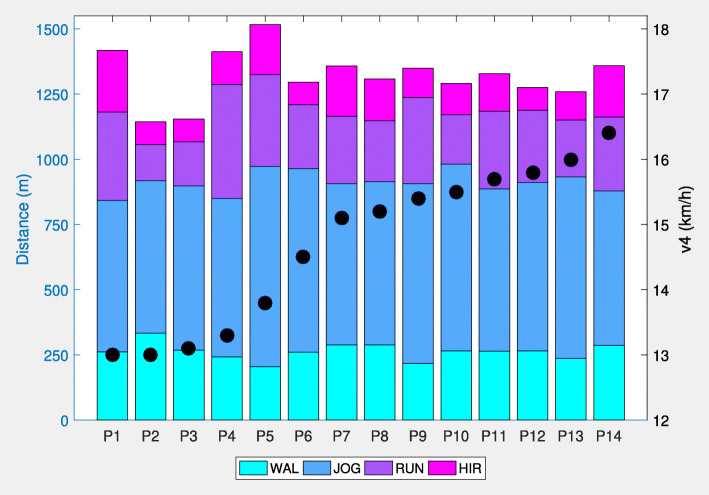
Distances covered in predefined speed zones (stacked bars) and velocity at a fixed blood lactate concentration of 4 mmol L^−1^ (black dots) in 14 male soccer players (P1–P14). WAL, walking (0.0–7.2 km/h); JOG, jogging (> 7.2–14.4 km/h); RUN, running distance (> 14.4–19.8 km/h); HIR, high-intensity running distance (> 19.8 km/h); *n*_acc_, number of accelerations (> 1.54 m s^−2^); ØHR, mean heart rate; HRR, heart rate recovery; v4, velocity at 4 mmol L^−1^ lactate threshold

In all players, v4 in the ITT ranged from 13.0 to 16.4 km/h (Fig. 2 and Table 1) with corresponding values for HR_v4_ from 88 to 94%. Furthermore, TD in the SSGs was between 1.14 and 1.52 km. While the major amount of this distance (59 to 80%) has been covered with velocities below 14.4 km/h (WAL + JOG), the smallest proportion (7 to 17%) was covered in the HIR zone. As expressed by CV, the highest interindividual variability was found for HIR (36%), followed by RUN (30%), WAL (12%), JOG (9%), and TD (8%). The total number of acceleration efforts above 1.54 m s^−2^ (*n*_acc_) was in the range of 27 to 63 with the average around 50. The internal load, as expressed by ØHR and L_SSGs_, was between 88 and 94%, or 8.5 and 17.7 mmol L^−1^, respectively. On average, the players needed 33.2 s (range, 14 to 58 s) to reduce their HR from 90 to 80% after the final SSGs bout. When looking at the relationships between the parameters investigated, ØHR was not associated with any other variable (Table 2). In contrast, a very large correlation with an explained variance of 84% was found between HRR and v4. In addition, TD was largely correlated with the distances covered in RUN and HIR zones. WAL showed a moderate inverse correlation with TD and all other speed zones, with the exception of HIR. Furthermore, moderate correlations were found for *n*_acc_ and HRR as well as for *n*_acc_ and v4. L_SSGs_ was moderately related to HRR and v4.

**Table 1:**
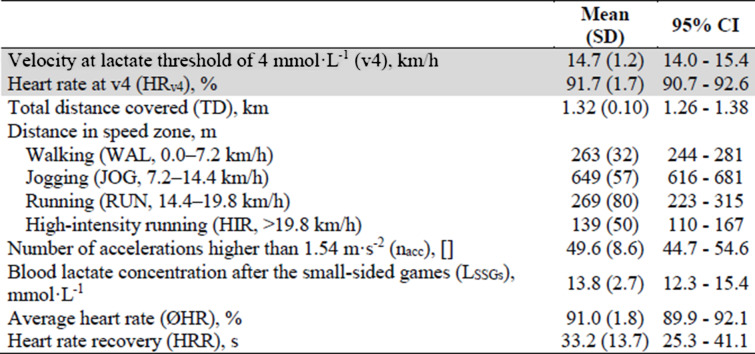
Main outcomes of the incremental treadmill test (highlighted in gray) and the small-sided games

Abbreviations: *SD* standard deviation, *CI*, confidence interval

**Table 2:**
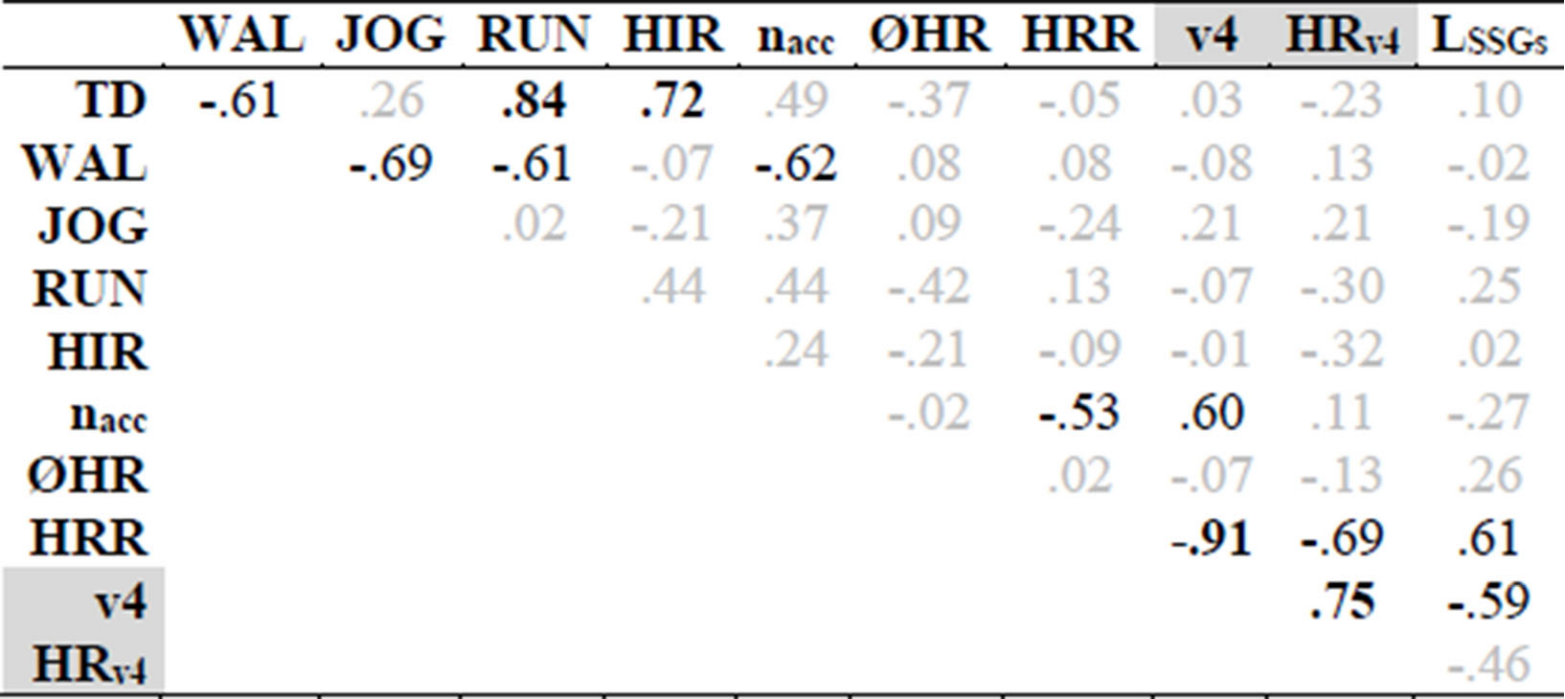
Pearson’s correlations of external and internal load parameters (outcomes of the incremental treadmill test are highlighted in gray)

When correlation is large (0.5–0.7), the correlation coefficient, *r*, is reported in black. Higher correlations (0.7–1.0, very large and almost perfect) are marked in bold

*TD* total distance, *WAL* walking, *JOG* jogging, *RUN* running distance, *HIR* high-intensity running distance, *n*_*acc*_ number of accelerations (> 1.54 m s^−2^), *ØHR* mean heart rate, *HRR* heart rate recovery, *v4* velocity at 4 mmol L^−1^ lactate threshold, *HRv4* heart rate at v4, *L*_*SSGs*_ blood lactate concentration after the small-sided games

## Discussion

The main result of the present study was a very large correlation (*r* = − .91) between HRR and v4, implying a link between the ability to recover from SSGs and the general aerobic endurance performance of semi-professional soccer players. Thus, besides being associated with endurance capacity, v4 may also help to evaluate soccer players’ specific ability to recover. Furthermore, the present investigation proved that soccer players with a higher v4 accumulated less lactate during SSGs (explained variance, 35%). Simultaneously, a better performance in the ITT was related with a higher number of accelerations (*n*_acc_) during SSGs (explained variance, 36%). Interestingly, TD and distances in predefined speed zones (WAL, JOG, RUN, HIR) did not show any relevant linear association to v4.

In a recent study, Schwesig et al. [11] investigated the differences in running velocities at lactate thresholds of 2, 4, and 6 mmol L^−1^ among male soccer players (*n* = 152; 3rd and 4th German league). Significant performance differences between playing positions were detected only between field players and goalkeepers. In comparison with the total sample of Schwesig et al. [11], the endurance of the investigated soccer players at v4 ranged between the 25th (14.4 km/h) and 50th percentile (15.0 km/h). Consequently, the aerobic endurance performance level of the recruited sample can be classified as normal to good. In addition, Altmann et al. [28] analyzed the physical capacity of soccer players from the 2nd German league utilizing a similar test design (start speed, 6 km/h; duration per speed level, 3 min; increment, 2 km/h). These authors found a slightly higher v4 (15.1 km/h) in field players (regardless of playing position) compared to the current study. For the 3rd league of Greece, Kalapotharakos et al. [10] showed a clearly lower endurance performance (v4, 12.3–13.7 km/h) using identical increments and speed level durations.

In the majority of research investigating the physiology of SSGs in soccer ØHR, BLC and the rating of perceived exertion (RPE) were used as indicators of exercise intensity [22, 23, 29]. In a representative study, Rampinini et al. [30] examined the effects of different modalities (e.g., number of players, pitch size, coach encouragement) on the exercise intensity in repeated bouts (3 × 4 min with 3 min recovery) of SSGs without goalkeepers. In the most physically intense variant (3 vs. 3 on a pitch of 18 × 30 m), RPE was 8.5 ± 0.4, BLC was 6.5 ± 1.5 mmol L^−1^, and ØHR was 91 ± 2%. A similar load protocol (6 × 2 min with 2 min recovery) to that used in the present study was applied by Köklü et al. [31]. Besides other forms, the authors investigated a 4 vs. 4 (without goalkeepers; game objective: keep ball possession; pitch dimensions, 25 × 32 m) and determined values of 5.2 ± 1.3, 7.9 ± 2.2 mmol L^−1^, and 85 ± 3% for RPE, BLC, and ØHR, respectively. Furthermore, the authors reported that around 77% of TD was covered with running velocities below 13 km/h. In comparison, the players in the current study covered only 61% of the distance at speeds below 13 km/h, indicating a clearly higher intensity, which in turn explains the greater ØHR and BLC (Table 1). BLC at a similar level as in the current study (11.4 ± 3.31 mmol L^−1^) has been reported by Castagna et al. [32] in repeated bouts (4 × 30 s with 150 s recovery) of 1 vs. 1 SSGs with mini-goals (1.5 × 2 m) played on a relatively large pitch (30 × 20 m). The SSGs were performed all-out; ergo, the players were told to cover as much distance as possible to create maximal effort (RPE, 8 ± 1). In total, the players covered a distance of 601 ± 54 m, resulting in an average velocity of about 18 km/h and corresponding to more than twice the mean velocity of our study (8.7 km/h). This high average velocity may have been due to Castagna et al. [32, 33] focus on the development of players’ extended sprint ability. For this purpose, the authors selected an exercise density of 300 m^2^ per player and a work to rest ratio of 1:5. Given the fact that all available studies on SSGs reported clearly lower values for BLC, the current study’s chosen load protocol appears to be particularly suited to provoke a very high BLC in a soccer-specific test situation. Thus, this also enables the evaluation of players’ anaerobic capacity that is an important precondition to cope with high-intensity phases during matches more frequently and for longer periods of time [32]. All things considered, the SSG protocol utilized in the present study caused a very high internal load for the players involved. Since the investigation has been carried out during the pre-season period, it is to be assumed that the fitness level was comparably low. For example, Rampinini et al. [30] collected their data from September to June (except for the period between December and January).

As can be seen in Fig. 2, the individual differences in TD were comparably small (CV < 8%), whereas the running performance was more variable among the players in the upper speed zones (RUN and HIR) resulting in CV values higher than 30%. This finding may represent divergent individual player profiles (continuous vs. explosive) or playing styles (playmaker vs. dribbler) that can only be affected to a limited extent by the modalities of the SSGs. Furthermore, the activity profiles of the players were completely independent from their individual aerobic performance. This fact becomes more notable, since the worst (P1) and the best player (P14) with respect to v4 showed nearly identical activity profiles. However, the acceleration efforts (*n*_acc_) showed a moderate linear association to v4. Considering that accelerating is more energetically demanding than moving at a constant velocity [34], better aerobic and anaerobic capabilities may enable players to execute accelerations more frequently within a defined timeframe. Moreover, peaks in the acceleration also reflect soccer-specific movements (i.e., starts, turns, directional changes) and thus maybe more relevant for the purpose of performance diagnostics in SSGs than distances in defined speed zones.

The ability to recover (as expressed by HRR) following a soccer-specific test (especially in SGGs) has not been investigated until now. Consequently, no evidence is available for the relationship between the ability to recover and endurance performance. Faster HRR is due to the faster reactivation of vagal activity [15]. Seiler et al. [35] pointed out that highly trained runners showed a higher vagal activity after intensive exercise over several hours compared to those with less running experience. Furthermore, individual resilience strongly affects the ability to recover. In the present study, an inverse association of v4 and L_SSGs_ was observed. This is in line with results reported after repeated-sprint exercise [36]. In a previous investigation, Buchheit et al. [36] concluded that an increased concentration of plasma metabolites of anaerobic metabolism causes a delay in post-exercise vagal reactivation, which also delays HRR.

The reader should be aware of the limitations of the current study. First of all, it has to be remarked that the here presented outcomes and relationships are based on only one observation among a relatively small sample of 14 players. Secondly, the study was conducted during the first week of pre-season preparation. Hence, different results might be expected during the competitive season when the physical fitness level of players may be improved. To avoid overinterpretation, the preliminary findings of the current study should be re-evaluated (external validation). The external validation should be performed with different samples and performance levels (e.g., 1st, 2nd, and 3rd leagues). Further, it should be noted that the evaluation of internal load and recovery was only at the cardiac level (HR, HRR). However, recovery and fatigue are multidimensional (e.g., metabolic, neural, hormonal) phenomena. Thus, in future studies, different outcomes (e.g., creatine kinase, urea, muscular stiffness) should also be considered.

## Conclusions

This study showed that a strong relationship exists between heart rate recovery after a standardized 4 vs. 4 SSG and the endurance performance in a laboratory setting among semi-professional soccer players. The distances covered in predefined speed zones, as well as total distance, were not linearly related to the v4. Although activity profiles were comparable between players, the number of accelerations was different and showed a moderate correlation to v4 and the heart rate recovery. The strongest relationship was detected between v4 and the heart rate reduction from 90 to 80% after the last bout of the small-sided games.

## Data Availability

The datasets used and analyzed during the current study are available from the corresponding author on reasonable request.
